# Comparative analysis of whole spine and sacroiliac joint magnetic resonance SPARCC scores in patients with SAPHO syndrome and ankylosing spondylitis

**DOI:** 10.3389/fimmu.2026.1803830

**Published:** 2026-06-04

**Authors:** Yi Shen, Xiaoli Gu, Jingchen Yang, Mi Zhou, Zhimin Lin, Rongsheng Wang, Jia Liu, Mengru Guo, Chen Li, Qi Zhu, Dongyi He

**Affiliations:** 1 Department of Rheumatology, Guanghua Hospital Affiliated to Shanghai University of Traditional Chinese Medicine, Shanghai, China; 2 Department of Rheumatology, Shanghai Guanghua Hospital of Integrative Medicine, Shanghai, China; 3 The Research Institute for Joint Diseases, Shanghai Academy of Traditional Chinese Medicine, Shanghai, China; 4 Department of Radiology, Guanghua Hospital Affiliated to Shanghai University of Traditional Chinese Medicine, Shanghai, China; 5 Department of Radiology, Shanghai Guanghua Hospital of Integrative Medicine, Shanghai, China; 6 School of nursing, Beijing University of Chinese Medicine, Beijing, China; 7 Department of Rheumatology, Third Affiliated Hospital, Beijing University of Chinese Medicine, Beijing, China

**Keywords:** ankylosing spondylitis, bone marrow edema (BME), SAPHO syndrome, SPARCC score, whole spine MRI

## Abstract

**Background:**

Ankylosing spondylitis (AS) and SAPHO syndrome both involve the spine and sacroiliac joints with bone marrow edema (BME) on MRI, which can lead to spinal structural damage. No previous study has compared BME characteristics between the two diseases.

**Methods:**

42 SAPHO and 60 AS patients with spinal or sacroiliac involvement underwent whole-spine and sacroiliac MRI. SPARCC scores for each spinal segment and sacroiliac joints, vertebral unit involvement frequency, and BME range, intensity, depth were compared. Correlation between SPARCC scores of the whole spine and sacroiliac joints and clinical indicators were analyzed in the two groups of patients.

**Results:**

SPARCC scores for all spinal segments and sacroiliac joints in AS patients were significantly higher than those in SAPHO syndrome patients (P < 0.01). Defining bone marrow edema as SPARCC score > 0, the positive rate of vertebral units in spinal segments and sacroiliac joints was higher in AS than in SAPHO syndrome (P < 0.05). The incidence of BME in the cervical, thoracic, and sacroiliac joints was higher in AS than in SAPHO syndrome (P<0.001), with no significant difference in the incidence of lumbar spine BME (P > 0.05); the incidence of bilateral sacroiliac joint edema also showed no intergroup difference (P > 0.05). Further analysis of the score composition in SPARCC-positive cases revealed: the extent scores for each spinal segment in AS patients were higher than those in the SAPHO group (*P* < 0.001), while there were no significant differences in intensity and depth scores between the two groups; in the sacroiliac joints, the extent score was higher in the AS group (*P* < 0.05), but the intensity score was higher in the SAPHO group (*P* < 0.05). Correlation analysis in AS and SAPHO patients showed: spinal SPARCC scores in AS patients were positively correlated with ESR, CRP, and ASDAS-CRP; spinal SPARCC scores in SAPHO patients were positively correlated with BASFI and BASMI. In subgroup analysis, AS patients showed positive correlations between spinal SPARCC scores and ASDAS-CRP at both thresholds (≥2, ≥3), and sacroiliac joint SPARCC scores were positively correlated with BASDAI at both thresholds; at threshold ≥3, sacroiliac joint SPARCC scores were also positively correlated with ASDAS-CRP. In SAPHO patients, at threshold ≥2, spinal SPARCC scores were positively correlated with VAS, BASFI, and BASMI; at threshold ≥3, spinal SPARCC scores were only positively correlated with BASFI, and sacroiliac joint SPARCC scores were positively correlated with ASDAS-CRP (all *P* < 0.05).

**Conclusions:**

SAPHO and AS show similar BME characteristics on MRI. SAPHO patients have a smaller extent of BME in spine and sacroiliac joints, whereas spinal BME may have a stronger correlation with their physical function. In SAPHO patients with axial involvement, the treatment experience of SPA may have certain reference value.

## Background

Synovitis, acne, pustulosis, hyperostosis, and osteitis (SAPHO) syndrome is a rare chronic clinical syndrome characterized by bone, joint, and skin inflammation (1). Spondyloarthritis (SpA) refers to a group of inflammatory diseases involving the medial and peripheral joints and sharing common features regarding pathogenesis, pathological changes, clinical manifestations, and imaging characteristics. Ankylosing spondylitis (AS) is a prototype of this disease group (2, 3). AS primarily involves the sacroiliac joints, spine, paraspinal soft tissues, and peripheral joints and may be accompanied by extra-articular manifestations, spinal deformity, and ankylosis in severe cases (4). Sacroiliac arthritis is a characteristic hallmark and early manifestation of AS. Furthermore, mesoaxial joint involvement is common in SAPHO syndrome. A retrospective analysis of SAPHO syndrome found spinal or sacroiliac joint involvement in 55.6% of the patients (5). Some studies have found spinal involvement in SAPHO syndrome in 32–52% of patients, manifested as vertebral osteosclerosis, osteophytes, paravertebral ossification, or vertebral collapse (6). Sacroiliac joint involvement occurs in approximately 13–52% of patients with SAPHO syndrome and manifests as sacroiliac arthritis, osteosclerosis, bone erosion, or ankylosis (7, 8). Both SAPHO syndrome and AS can present with back pain, and elevated blood sedimentation rate (ESR) and C-reactive protein (CRP) levels, and 30% of patients with SAPHO syndrome are HLA-B27 positive (9). SAPHO syndrome and SpA have overlapping osteoarticular, imaging, inflammatory marker, and genetic features (10). However, SAPHO is generally regarded as a distinct disease entity. As an autoinflammatory syndrome, its core mechanism involves excessive activation of innate immunity, leading to elevated levels of various pro-inflammatory cytokines, such as tumor necrosis factor (TNF)-α, interleukin (IL)-1, IL-8, IL-17, and IL-18 (11). Moreover, several studies have isolated pathogens such as *Cutibacterium acnes*, *Staphylococcus aureus*, *Haemophilus parainfluenzae*, and *Actinomyces* from SAPHO osteitic lesions (12), suggesting that infectious agents may trigger an exaggerated inflammatory response, thereby potentially classifying SAPHO syndrome under the category of “reactive osteitis.” (13) The differences between skeletal inflammation in SAPHO and that in SPA have not been thoroughly investigated.

Magnetic resonance imaging (MRI) can detect active inflammatory lesions, such as osteitis (showing as bone marrow edema, BME), and is suitable for early diagnosis and assessment of disease activity and treatment efficacy. Bone marrow inflammation is the most common lesion during the active phase of SpA, manifesting as BME on MRI; it can be observed in the vertebral horns, vertebral tuberosity, sacroiliac joints, and sternoclavicular joints. Bone marrow inflammation leads to bone and articular cartilage erosion, subsequently causing reparative changes and structural damage. Vertebral BME in SpA can be secondary to fat deposition, which promotes the formation of new bone, such as vertebral rim osteophyte formation and spinal ligament calcification (14), resulting in a reduced range of spinal motion. Abnormal bone formation is a major cause of spinal ankylosis and deformity (15). Impaired spinal mobility is common in AS, persisting up to 10 years, and reduced lateral spinal flexion is particularly associated with further spinal dyskinesia in the later disease course (16). Reduced spinal mobility reduces the ability to work and live and impacts mental health (17). SAPHO syndrome also involves inflammation-driven damage to bone structure and may trigger ligamentous attachment point inflammation, causing adjacent bone edema, destruction, and sclerosis (18). SAPHO syndrome presents with recurrent, relapsing, and remitting pathological processes, with coexisting inflammatory destruction and vertebral body margin sclerosis. Rapid inflammatory destruction can lead to vertebral collapse and fractures (19).

Given the significant impact of spinal inflammation on structural damage and overall prognosis, accurate assessment of the degree of vertebral bone marrow inflammation is essential for evaluating disease activity and selecting appropriate treatment strategies. Significant BME is observed on whole-spine and sacroiliac joint MRI in patients with SAPHO syndrome and AS (20–22); however, no studies have compared the extent, distribution, and characteristics of BME between the two diseases. In this study, we used a modified SPARCC (Spondyloarthritis Research Consortium of Canada) scoring system to compare the distribution and characteristics of BME in the spine and sacroiliac joints between patients with AS and those with SAPHO syndrome, aiming to identify associations between these two diseases and provide a basis for selecting treatment options for SAPHO syndrome.

## Methods

### Study population

From April 2020 to April 2023, 60 patients with AS and 42 patients with SAPHO syndrome were recruited from Guanghua Hospital affiliated with Shanghai University of Traditional Chinese Medicine. Patients with AS fulfilled the New York Criteria for AS revised in 1984 (23), and those with SAPHO syndrome fulfilled the diagnostic criteria for SAPHO syndrome proposed by Kahn in 1994 and revised in 2003 (24). All SAPHO patients included in this study had spinal and sacroiliac joint involvement. Involvement was defined as the coexistence of related clinical symptoms and imaging evidence. Clinical symptoms referred to pain in the back (buttock, lumbar or thoracic spine) or cervical region, with or without limited range of motion, lasting for more than one month. Imaging evidence referred to at least one positive finding in the spine or sacroiliac joints on bone scintigraphy, CT, or MRI (5). The research project was approved by the ethical committees of Shanghai Guanghua Hospital of Integrative Medicine.

### Imaging protocol

All patients underwent whole-spine and sacroiliac joint MRI using a SIEMENS 1.5TMR scanner equipped with a body coil. The sagittal position was selected for the spine and the transverse axial and oblique coronal positions for the sacroiliac joints. The scanning sequences included conventional T1WI and T2WI scans and a fast-inversion recovery T2WI fat-suppression sequence. The matrix size was 256×256, and the layer thickness was 4 mm.

### Image analysis

The MRI lesions were scored using the SPARCC (25) scoring system by two musculoskeletal radiologists with more than 10 years of experience. All images were digitized and anonymized in a standardized manner, and then independently and retrospectively assessed and scored by two radiologists. The reviewers were blinded to the chronological order of the imaging data, patient information, clinical diagnoses, and other examination results.The traditional SPARCC scoring system selects the six vertebral units with the most severe inflammatory lesions to score the extent, intensity, and depth of BME, with a total score of up to 108 points for the six units. This study used the revised SPARCC score, comprising the sum of the scores of all involved vertebral units, with a total score of 414 points (26). The SPARCC scoring system for the sacroiliac joints assigns a total score of 12 points to the sacroiliac joints bilaterally, with a maximum possible total score of 72. Vertebral units and sacroiliac joints with SPARCC scores >0 were defined as having positive SPARCC scores. BME was defined as a vertebral unit or sacroiliac lesion with a SPARCC score >0.

### Clinical assessment

Clinical data, including age, sex, age at onset, and disease duration were collected. Assessment results, including those of the visual analog scale (VAS) for pain (0-10), Bath AS Disease Activity Index (BASDAI) (27), Bath AS Functional Index (BASFI) (28), patients’ assessment of their overall disease status, Ankylosing Spondylitis Disease Activity Score (ASDAS) (29), Bath AS Measurement Index (BASMI) (30), and ultra-sensitive CRP and ESR laboratory tests, were collected.

### Statistical analysis

SPSS version 26.0 (SPSS, Chicago, IL, USA) was used to analyze the data. For measurable variables, the mean and standard deviation were presented if the data were normally distributed. The intraclass correlation coefficient (ICC) was used to assess inter-reader agreement. Multiple linear regression analysis was applied to investigate the effects of age, disease duration, and sex on SPARCC scores of the spine and sacroiliac joints. The independent sample *t-*test was used for inter-group comparison and the independent sample Mann–Whitney U-test was used for inter-group comparison of non-normally distributed data. For immeasurable variables, percentages were used to present the data, and the chi-square test was used to compare the differences. Correlation analysis was conducted using Spearman’s correlation analysis. Statistical significance was set at *P* < 0.05.

## Results

### Assessment of reader agreement for SPARCC score measurements

The two readers demonstrated excellent agreement on SPARCC scores for the spine (cervical, thoracic, lumbar) and sacroiliac joints in both AS and SAPHO patients, with ICC values all exceeding 0.90 (see **Tables 1**, **2**).

**Table 1:** Results of inter-reader consistency test for AS SPARCC scores [median (interquartile range)].

Assessment items	Reader 1	Reader 2	ICC	95%CI
spine(0-414)	24.50(8.00, 46.00)	25.00(8.00, 45.00)	0.988	0.979-0.993
cervical vertebrae (0-108)	2.00(0, 9.75)	3.00(0, 10.75)	0.996	0.994-0.998
thoracic vertebrae (0-216)	11.50(3.00, 28.75)	12.50(2.25, 26.75)	0.988	0.981-0.993
lumbar vertebrae (0-90)	2.50(0, 13.75)	3.00(0, 11.00)	0.941	0.904-0.965
sacroiliac joints (0-72)	7.00(0, 16.50)	6.00(0, 15.75)	0.952	0.920-0.971

**Table 2:** Results of inter-reader consistency test for SAPHO SPARCC scores [median (interquartile range)].

Assessment items	Reader 1	Reader 2	ICC	95%CI
spine(0-414)	5.00(0, 14.25)	6.50(0.75, 16.00)	0.947	0.903-0.971
cervical vertebrae (0-108)	0(0, 0)	0(0, 1.00)	0.936	0.884-0.965
thoracic vertebrae (0-216)	11.50(3.00, 28.75)	12.50(2.25, 26.75)	0.988	0.981-0.993
lumbar vertebrae (0-90)	2.00(0, 5.00)	2.00(0, 6.00)	0.939	0.889-0.967
sacroiliac joints (0-72)	0(0, 3.50)	0(0, 4.00)	0.970	0.945-0.984

### Demographic and clinical characteristics

60 patients with AS (19 women and 41 men, 19–69 years) and 42 with SAPHO syndrome (34 women and 8 men, 17–67 years) were included. Age was not statistically different between the two groups. Patients with AS were primarily men, whereas those with SAPHO syndrome were primarily women. Patients with AS had longer disease durations, earlier ages of onset, and higher CRP levels, whereas those with SAPHO syndrome had higher ESR levels and VAS scores. Differences in BASDAI, BASFI, and ASDAS-CRP scores were not statistically significant, suggesting a lack of difference in disease activity between the two groups. The BASMI was higher in AS than in SAPHO syndrome (**Table 3**). Based on the ASDAS-CRP criteria, the patients in the two groups were categorized into four disease activity subgroups. The results showed no significant differences in the number and proportion of patients across disease activity levels between the two groups (χ² = 0.437, *P* > 0.05), as shown in **Table 4**. Furthermore, there were no statistically significant differences in ASDAS scores among the disease activity levels (*P* > 0.05), as presented in **Table 5**. These findings indicate a high degree of comparability between the two groups in terms of disease activity. We performed multiple linear regression analysis to evaluate the contributions of age, disease duration, and sex to SPARCC scores of the spine and sacroiliac joints in patients with AS and SAPHO syndrome, respectively. The results showed that for AS patients, the standardized coefficients (β) of age, sex, and disease duration for spinal SPARCC scores were 0.057, -0.203, and 0.010, with p-values of 0.714, 0.159, and 0.951, respectively; for sacroiliac joint SPARCC scores, the β values were -0.558, 0.185, and -0.154, with p-values of 0.579, 0.854, and 0.330, respectively; the variance inflation factors (VIF) were 1.386, 1.175, and 1.437, respectively. For SAPHO patients, the β values of the three variables for spinal SPARCC scores were 0.234, -0.201, and -0.004, with p-values of 0.152, 0.203, and 0.983, respectively; for sacroiliac joint SPARCC scores, the β values were 0.138, 0.202, and 0.125, with p-values of 0.403, 0.209, and 0.447, respectively; the VIF values were 1.091, 1.020, and 1.090, respectively. None of the regression coefficients were statistically significant in either patient group (*p* > 0.05), and all VIF values were less than 2, indicating no significant multicollinearity. These findings suggest that age, disease duration, and sex do not independently influence spinal or sacroiliac joint SPARCC scores in AS or SAPHO patients.

**Table 3:** Demographic and clinical characteristics.

Parameter	AS	SAPHO syndrome	*P* - value
Sex (male/female)	45/15	8/34	0.000
Age( ± *s*)/years	43.23 ± 11.76	46.93 ± 12.10	0.315
Disease duration( ± *s*)/months	154.63 ± 93.49	89.43 ± 69.07	0.001
Age of onset/years	30.35 ± 10.27	38.19 ± 11.8	0.001
CRP	11.27 ± 12.75	6.29 ± 8.61	0.030
ESR	20.79 ± 18.58	33.48 ± 27.85	0.007
VAS	3.72 ± 2.21	5.30 ± 1.80	0.000
BASDAI	2.91 ± 1.66	3.49 ± 1.78	0.091
BASFI	1.95 ± 1.38	2.09 ± 1.44	0.626
ASDAS-CRP	2.28 ± 0.93	2.31 ± 0.99	0.855
BASMI	3.65 ± 2.04	1.14 ± 1.34	0.000

AS, Ankylosing spondylitis; SAPHO, synovitis, acne, pustulosis, hyperostosis, and osteitis; CRP, C-reactive protein; ESR, erythrocyte sedimentation rate; VAS, Visual Analogue Scale; BASDAI, Bath Ankylosing Spondylitis Disease Activity Index; BASFI, Bath Ankylosing Spondylitis Functional Index; ASDAS, Ankylosing Spondylitis Disease Activity Score; BASMI, Bath Ankylosing Spondylitis Measurement Index.

**Table 4:** Disease activity grading of patients in the two groups (n/%).

ASDAS grading	Inactive disease	Low disease activity	High disease activity	Very high disease activity
AS(n=60)	7(11.67%)	23(37.10%)	23(37.10%)	7(11.29%)
SAPHO(n=42)	6(14.29%)	14(33.33%)	16(38.10%)	6(14.29%)

**Table 5:** Comparison of ASDAS-CRP between the two groups (Mean ± SD).

ASDAS grading	Inactive disease	Low disease activity	High disease activity	Very high disease activity
AS(n=60)	0.86 ± 0.23	1.72 ± 0.45	2.78 ± 0.36	3.40 ± 0.39
SAPHO(n=42)	0.85 ± 0.35	1.75 ± 0.22	2.74 ± 0.38	3.95 ± 0.43

### Comparison of SPARCC scores and spinal and sacroiliac joint involvement

When applying the revised SPARCC scoring system to assess magnetic resonance imaging (MRI) findings of the entire spine, the AS patient group exhibited significantly higher SPARCC scores across all spinal segments compared to the SAPHO patient group (*P*< 0.01). Similarly, SPARCC scores for the sacroiliac joints were also markedly elevated in AS patients relative to those with SAPHO syndrome (*P* < 0.01), as presented in **Table 6**.

The 23 vertebral units of the entire spine were divided into three segments: the cervical (DVU1-5), thoracic (DVU6-17), and lumbar (DVU18-23) vertebrae. Overall, 1380 AS vertebral units, including 300 cervical, 720 thoracic, and 360 lumbar vertebrae; and 966 SAPHO vertebral units, including 210 cervical, 504 thoracic, and 252 lumbar vertebrae, were analyzed. The proportion of vertebral units with positive SPARCC scores in spinal segments and sacroiliac joints was higher in AS than in SAPHO syndrome (*P* < 0.05). Whole-spine MRI showed that the proportions of vertebral units with cervical, thoracic, and lumbar BME in SAPHO syndrome and AS were 5.0% to 25.3%, 9.38% to 28.6%, and 17.08% to 23.6%, respectively. The frequencies of sacroiliac joint BME were 27.5% and 53.3%, respectively (**Table 6**).

**Table 6:** SPARCC scores and spinal and sacroiliac joint involvement comparison between AS and SAPHO syndrome.

Characters	AS (n=60)			SAPHO syndrome(n=42)		
	Score	Positive units	Positive rate (%)	Score	Positive units	Positive rate (%)
Cervical vertebrae	8.20 ± 14.50	76/300	25.3	0.79 ± 2.65**	10/200	5.0**
Thoracic vertebrae	20.93 ± 27.13	206/720	28.6	4.26 ± 6.24**	45/480	9.38**
Lumbar vertebrae	7.73 ± 10.31	85/360	23.6	4.02 ± 6.94*	41/240	17.08*
Sacroiliac joint	9.6 ± 10.54	64/120	53.3	5.05 ± 11.25**	22/80	27.5**
Spine	36.87 ± 43.76	367/1380	26.6	9.07 ± 10.95**	96/920	10.43**

*P < 0.05, **P < 0.01 when compared to cases with positive AS SPARCC scores and positive rate.

AS, Ankylosing spondylitis; SAPHO, synovitis, acne, pustulosis, hyperostosis, and osteitis; SPARCC, Spondylarthritis Research Consortium of Canada.

### Associations between SPARCC scores and clinical measurements

We investigated the relationship between changes in SPARCC scores and seven clinical measurements: ESR, CRP, VAS, BASDAI, BASFI, ASDAS, and BASMI, which represent disease activity or functional impairment. Spearman’s correlation analysis showed that the spine score positively correlated with ESR, CRP, and ASDAS-CRP levels in AS (*P* < 0.05), and the spine plus sacroiliac score positively correlated with ASDAS-CRP (*P* < 0.05) (**Table 7**). The SPARCC spine score, and the spine plus sacroiliac score, positively correlated with BASFI and BASMI in SAPHO syndrome (*P* < 0.05) (**Table 8**).

**Table 7:** Correlation of SPARCC scores with clinical measurements in patients with AS(R Value*, P* Value).

Clinical measurements	Spine		Sacroiliac		Spine plus sacroiliac	
	R	*P*	R	*P*	R	*P*
CRP	.256^*^	.048	-.015	.911	.235	.071
ESR	.258*	.047	.099	.453	.232	.075
VAS	.224	.085	.207	.113	.250	.054
BASDAI	.236	.069	-.069	.598	.245	.059
BASFI	.114	.387	-.042	.748	.106	.420
ASDAS	.341^**^	.008	.029	.826	.334^**^	.009
BASMI	.239	.065	.126	.337	.080	.543

SPARCC, Spondylarthritis Research Consortium of Canada; AS, Ankylosing spondylitis; CRP, C-reactive protein; ESR, erythrocyte sedimentation rate; VAS, Visual Analogue Scale; BASDAI, Bath Ankylosing Spondylitis Disease Activity Index; BASFI, Bath Ankylosing Spondylitis Functional Index; ASDAS, Ankylosing Spondylitis Disease Activity Score; BASMI, Bath Ankylosing Spondylitis Measurement Index. Correlations between clinical measurements of AS patients and SPARCC scores of the spine or sacroiliac joints, *P < 0.05, **P < 0.01.

**Table 8:** Correlation of SPARCC scores with clinical measurements in patients with SAPHO syndrome(R Value*, P* Value).

Clinical measurements	Spine		Sacroiliac		Spine plus sacroiliac	
	R	*P*	R	*P*	R	*P*
CRP	.102	.532	.2250	.163	.122	.453
ESR	.019	.909	.096	.558	-.003	.984
VAS	192	.235	-.019	.908	.223	.166
BASDAI	.143	.387	.015	.924	.080	.625
BASFI	.432**	.005	.126	.439	.401*	.010
ASDAS	.177	.274	-.059	.716	.075	.644
BASMI	.472**	.002	.066	.686	.351*	.026

Correlations between clinical measurements of SAPHO patients and SPARCC scores of the spine or sacroiliac joints, *P < 0.05, **P < 0.01.

Patients were divided into two subgroups for statistical analysis using SPARCC score thresholds of ≥2 and ≥3, respectively. The results showed that among AS patients, 56 had a spinal SPARCC score ≥2 and 52 had a score ≥3; 39 had a sacroiliac joint SPARCC score ≥2 and 37 had a score ≥3. Among patients with SAPHO syndrome, 29 had a spinal SPARCC score ≥2 and 26 had a score ≥3; 13 had a sacroiliac joint SPARCC score ≥2 and 11 had a score ≥3. Further correlation analysis between SPARCC scores and disease activity as well as functional indices revealed the following: AS patients: The spinal SPARCC score was positively correlated with ASDAS-CRP level under both thresholds (all *P* < 0.05). The sacroiliac joint SPARCC score was positively correlated with BASDAI (*P* < 0.05). Additionally, when the SPARCC score was ≥3, the sacroiliac joint SPARCC score was also positively correlated with ASDAS-CRP level (*P* < 0.05). See **Table 9** for details. SAPHO syndrome patients: When the SPARCC score was ≥2, the spinal SPARCC score was positively correlated with VAS score, BASFI, and BASMI (all *P* < 0.05). When the SPARCC score was ≥3, the spinal SPARCC score was positively correlated with BASFI, and the sacroiliac joint SPARCC score was positively correlated with ASDAS-CRP level (all *P* < 0.05). See **Table 10** for details.

**Table 9:** Correlation of SPARCC scores with clinical measurements in patients with AS(R Value, P Value) (SPARCC≥2, ≥3).

Clinical measurements	Spine(n=56)		Sacroiliac(n=39)		Spine(n=52)		Sacroiliac(n=37)	
	R	*P*	R	*P*	R	*P*	R	*P*
CRP	.137	.314	.125	.448	.154	.275	.195	.247
ESR	.182	.179	.194	.236	.123	.385	.314	.058
VAS	.166	.221	.219	.180	.203	.149	.271	.105
BASDAI	.236	.080	.394^*^	.013	.173	.219	.379*	.021
BASFI	.073	.595	.140	.394	.007	.950	.152	.370
ASDAS	.299^*^	.025	.308	.056	.278^*^	.046	.356^*^	.031
BASMI	.150	.270	-.277	.088	.113	.424	-.219	.193

Correlations between clinical measurements of AS patients and SPARCC scores of the spine or sacroiliac joints, *P < 0.05.

**Table 10:** Correlation of SPARCC scores with clinical measurements in patients with SAPHO syndrome (R Value*, P* Value) (SPARCC≥2, ≥3).

Clinical measurements	Spine(n=29)		Sacroiliac(n=13)		Spine(n=26)		Sacroiliac(n=11)	
	R	*P*	R	*P*	R	*P*	R	*P*
CRP	.272	.153	.050	.872	.287	.154	-.282	.401
ESR	.139	.473	-.417	.156	.148	.469	-.555	.077
VAS	.373^*^	.047*	.129	.676	.212	.299	-.388	.238
BASDAI	.203	.290	-.149	.628	.343	.086	-.674^*^	.023
BASFI	.616^**^	.000	.145	.637	.592^**^	.001	-.310	.354
ASDAS	.222	.248	-.324	.280	.214	.295	-.596	.053
BASMI	.444^*^	.016	-.004	.989	.359	.071	.009	.978

*P < 0.05, **P < 0.01.

SPARCC, Spondylarthritis Research Consortium of Canada; SAPHO, synovitis, acne, pustulosis, hyperostosis, and osteitis; CRP, C-reactive protein; ESR, erythrocyte sedimentation rate; VAS, Visual Analogue Scale; BASDAI, Bath Ankylosing Spondylitis Disease Activity Index; BASFI, Bath Ankylosing Spondylitis Functional Index; ASDAS, Ankylosing Spondylitis Disease Activity Score; BASMI, Bath Ankylosing Spondylitis Measurement Index. Correlations between clinical measurements of SAPHO patients and SPARCC scores of the spine or sacroiliac joints, *P < 0.05, **P < 0.01.

### Comparison of the incidence of BME in the spine segments and sacroiliac joints

Cervical spine BME was observed in eight (8/42, 19.0%) and 31(31/60, 51.67%) patients with SAPHO syndrome and AS, respectively. Thoracic spine BME was observed in 21 (21/42,50%) and 50 (50/60, 83.33%) patients with SAPHO syndrome and AS, respectively. Lumbar spine bone marrow edema was observed in 22 (22/42, 52.38%) and 35(34/60, 56.67%) patients with SAPHO syndrome and AS, respectively. The incidence of sacroiliac joint BME were 35.71% (15/42) and 65.0% (39/60) in SAPHO syndrome and AS, respectively. The incidence of BME in the cervical, thoracic, and sacroiliac joints was higher in AS than in SAPHO syndrome (*P* < 0.001), with no significant difference in the incidence of lumbar spine BME (*P*>0.05). The incidence of sacroiliac bilateral BME was 66.67% and 53.33% in AS and SAPHO syndrome, respectively, with no significant difference between the two groups (*P*>0.05).

### Differences in SPARCC score categories

We compared the range, intensity, and depth of SPARCC scores for each spinal segment in cases with positive SPARCC scores between the two groups. The range scores of all spinal segments were higher in AS than in SAPHO syndrome (*P* < 0.001), with no significant differences in intensity and depth scores between the two groups (*P*>0.05) (**Table 11**). The range scores of the sacroiliac joints were higher in AS than in SAPHO syndrome (*P* < 0.05), whereas the intensity scores were higher in SAPHO syndrome than in AS (*P* < 0.05; **Table 11**).

**Table 11:** Comparison of the range, intensity, and depth of spinal and sacroiliac joint SPARCC scores between AS and SAPHO syndrome.

SPARCC scores	Cervical vertebrae		Thoracic vertebrae		Lumbar vertebrae		Sacroiliac joints	
	AS(n=31)	SAPHO(n=8)	AS(n=50)	SAPHO(n=21)	AS(n=34)	SAPHO(n=22)	AS(n=39)	SAPHO(n=15)
Range	13.52 ± 14.76	3.0 ± 2.62**	21.26 ± 23.15	6.1 ± 4.09**	11.21 ± 8.21	5.36 ± 4.39*	12.49 ± 7.5	8.47 ± 8.78*
Intensity	0.94 ± 1.99	0.38 ± 1.06	1.30 ± 3.25	1.24 ± 1.78	0.82 ± 1.51	0.95 ± 1.86	0.74 ± 1.58	2.80 ± 3.51*
Depth	1.42 ± 2.54	0.75 ± 1.75	2.56 ± 4.03	1.19 ± 1.60	1.62 ± 2.24	1.36 ± 2.44	1.69 ± 2.34	2.87 ± 3.60

*P < 0.05, **P < 0.01 when compared to cases with positive AS SPARCC scores.

AS, Ankylosing spondylitis; SAPHO, synovitis, acne, pustulosis, hyperostosis, and osteitis; SPARCC, Spondylarthritis Research Consortium of Canada.

## Discussion

BME is the most prominent inflammatory lesion in AS and SAPHO syndrome. The inflammatory process in the AS spine usually begins at the anterior vertebral horn (Romanus lesion) and extends toward the endplates, anterior cortex, or both (31, 32). Romanus lesions indicate active spinal inflammation (33). Vertebral corners also constitute the main site of spinal involvement in SAPHO syndrome, particularly in the thoracic spine, where MRI demonstrates a BME signal or contrast enhancement (34) similar to that of anterior spinal horn lesions in AS (35). In addition to BME, fat deposits (100%), bone destruction (75%), and osteosclerosis (79.2%) were observed on the MRI of patients with SAPHO syndrome (36). BME of the sacroiliac joints in AS occurs in the medullary cavity below the articular surface and slightly more frequently on the iliac side, with limited small or diffuse large patches of abnormal signals. BME was observed in the neighboring bones, and the BME signal manifested as point-like and piecemeal abnormal signals under the articular surface with unclear borders (37).

The SPARCC score is currently the most widely used MRI scoring system and accurately evaluates the severity of BME in the spine and the sacroiliac joints in AS. Spinal and sacroiliac SPARCC scores positively correlate with AS disease activity indicators (38). Several clinical trials have reported significant improvements in spinal or sacroiliac SPARCC scores in patients with AS treated with biologics (39, 40). The total spinal and sacroiliac SPARCC score predicted etanercept efficacy in patients with AS, with correlations between SPARCC score improvements and ASDAS, BASDAI, CRP, and ESR improvements after etanercept treatment. The application of the SPARCC score to assess the effectiveness and significance of etanercept treatment was consistent with the clinical efficacy evaluation index ASAS40 (41). The revised SPARCC scoring system records the sum of the range, intensity, and depth scores of all affected spinal vertebral units and can more comprehensively and objectively reflect spinal inflammation in AS and SAPHO syndrome. Xu et al. applied the revised SPARCC semiquantitative scoring system for MRI evaluation and showed that the total MRI score had a strong linear correlation with the CRP-based ankylosing spondylitis activity score and CRP level (26). The total SPARCC score continued to improve after 12 months of treatment in patients with SAPHO syndrome, and the revised SPARCC score for spinal joints was the largest contributor to the total score, suggesting that the revised SPARCC score can assess the extensive radiologic damage, reflect disease activity, and serve as an important index for clinical efficacy evaluation in SAPHO syndrome (42). However, the revised SPARCC scoring system is not widely used in clinical practice. In this study, we used the revised SPARCC scoring system for the first time to comprehensively compare the differences in the extent, distribution, and intensity of spinal BME between patients with AS and SAPHO syndrome and to explore correlations between SPARCC scores and disease activity and functional impairment scores. The results showed that the spinal SPARCC score in AS patients was positively correlated with ASDAS-CRP levels at various thresholds, and the sacroiliac joint SPARCC score was positively correlated with BASDAI. When the SPARCC score was ≥3, the sacroiliac joint SPARCC score was also positively correlated with ASDAS-CRP levels. This fully confirms that bone marrow edema is closely associated with AS disease activity, and the correlation with spinal BME is more pronounced.

We found that the entire spine and sacroiliac joint SPARCC scores were higher in patients with AS compared to those with SAPHO syndrome, with the differences being more pronounced in the cervical and thoracic regions. Moreover, the percentage of SPARCC-positive vertebral units in each segment of the spine was higher in AS than in SAPHO syndrome. Further comparison of the range, intensity, and depth of SPARCC scores for each spinal segment between the two groups with positive SPARCC scores revealed that the range scores for each spinal segment were higher in AS than in SAPHO syndrome, with no significant differences in the intensity and depth scores between the two groups. Our results showed that the extent of BME in both the spine and sacroiliac bone was lower in SAPHO syndrome than in AS. Zhang et al. (43) compared the imaging characteristics of 28 patients with SAPHO syndrome and 44 patients with SpA and found that the prevalence of BME in the anterior horn of the spine in patients with SAPHO syndrome was 50%, which was lower than that in patients with SpA (75%), in line with the findings of this study.

Our study shows that in SAPHO syndrome, the SPARCC spinal score is positively correlated with BASFI and BASMI, with a particularly stronger correlation with BASFI. BASFI and BASMI are commonly used to measure physical function in patients with SpA. We explain the association between spinal inflammation and physical function in SAPHO syndrome from the following aspects. First, 80.3% of cases of vertebral body involvement in SAPHO syndrome show continuous involvement of the anastomosis (26), which can significantly affect the spinal range of motion of patients. Second, in addition to BME, MRI findings in SAPHO syndrome are often accompanied by other lesions, such as focal bone erosion and endplate inflammation. A retrospective evaluation of spinal MRI in 12 SAPHO syndrome patients with spinal involvement revealed that 96% of patients had at least one lesion with erosion of the anterior vertebral corner. Prevertebral soft tissue thickening was observed in 33% of patients (44). Disc involvement in SAPHO syndrome occurs in approximately 9–32% of patients (45), a higher percentage than that in AS (4.5%) (46), and its imaging features are similar to Andersson lesions in AS. Furthermore, the frequency of spinal bone erosion in SAPHO syndrome (75%) is significantly higher than that in SpA (36.4%), and the frequencies of erosion of the intervertebral discs, endplates, anterior chest wall, and paravertebral soft tissues are also higher in SAPHO syndrome than in SpA (47).In summary, patients with SAPHO syndrome tend to have coexisting inflammatory and structural lesions, resulting in a more severe impact on somatic function. This also suggests that patients with SAPHO syndrome with spinal BME may need more aggressive drug treatment measures to delay structural damage and improve long-term prognosis.

The sacroiliac joint is a common site of disease in SAPHO syndrome, manifesting as sacroiliac arthritis, osteosclerosis, bone erosion, and ankylosis (8, 9). However, the incidence of BME in the sacroiliac joints has rarely been reported. In our study, the frequency of sacroiliac joint BME in SAPHO syndrome was 27.5%, which was lower than that in AS (53.3%), and the percentage of sacroiliac joints with positive SPARCC in SAPHO syndrome was (35.71%, 15/42), which was also lower than that in AS (65.0%, 39/60). Nevertheless, other studies have suggested that sacroiliac joint involvement in SAPHO syndrome is more often bilateral, predominantly sacral, coexisting with old and new lesions, and less likely to cause joint ankylosis (37, 47). This study showed that the rate of bilateral sacroiliac involvement was 66.67% in patients with AS and 53.33% in patients with SAPHO syndrome, with no significant difference between the two groups. We found that the SPARCC intensity scores were significantly higher in SAPHO syndrome than in AS, which may be attributed to the pathological mechanisms underlying the two diseases. We found that the SPARCC intensity scores were significantly higher in SAPHO syndrome than in AS, which may be attributed to the pathological mechanisms underlying the two diseases. Pathological changes in AS show cellular infiltration of the synovial and subchondral bone marrow lymphocytes, macrophages, and plasma cells. In contrast, bone tissue biopsies in patients with SAPHO syndrome showed fibrous tissue hyperplasia or ossification in addition to infiltration of lymphocytes or plasma cells (48). Patients with SAPHO syndrome often present with a mixture of erosive sacroiliac arthritis and extensive osteosclerosis of the adjacent ilium or sacrum (49). These pathological characteristics may explain the higher signal intensity observed on T2-weighted fat-suppressed (T2FS) or STIR sequences in patients with SAPHO syndrome compared to those with AS on MRI.

This study had some limitations. First, this study primarily evaluated BME manifestations in the whole spine and sacroiliac joints of patients with SAPHO syndrome and AS, and structural lesions, such as bone erosion and endplate inflammation, were not explored. BME can objectively indicate the active inflammatory state of the disease; nevertheless, it does not reflect the complete disease picture. Future studies should compare other lesions, such as steatosis, intervertebral disc lesions, bone erosion, and ligamentous ossification, on MRI to comprehensively evaluate the imaging features and points of differentiation between SAPHO syndrome and AS. Second, the matching of disease activity between the two groups of patients enrolled in this study was not entirely consistent. In this study, patients with AS and SAPHO syndrome were rigorously matched for ASDAS CRP at enrollment, and there were no statistically significant differences in BASDAI or BASFI scores between the two groups, indicating a certain degree of comparability in disease activity. Multiple linear regression analysis showed that age, disease duration, and sex could not independently predict SPARCC scores. However, differences remained between the two groups in ESR, CRP, VAS score, and BASMI, which represent important confounding factors that were not fully controlled for by the current regression analysis. Future studies should expand the sample size, recruit cases from larger patient populations, comprehensively screen and match for multiple factors including inflammatory markers, disease activity, functional status, and structural damage, and establish prospective cohorts to enable more rigorous comparative analyses.Finally, due to the current lack of recognized disease activity and functional assessment tools for SAPHO syndrome, this study had to use assessment indicators relevant to AS, such as BASDAI, ASDAS, BASFI, and BASMI, to evaluate spinal activity in patients with SAPHO. Given that all SAPHO patients included in this study had spinal or sacroiliac joint involvement, the use of these indicators is to some extent feasible. However, it should be noted that these assessment tools were developed and validated specifically in patients with axial spondyloarthritis, and their construct validity in SAPHO syndrome—where the disease burden also involves the anterior chest wall, skin, and peripheral skeletal structures—has not been confirmed. Specifically: the extent of anterior chest wall involvement, which is common in SAPHO patients, is difficult to accurately quantify, and pain or swelling in peripheral joints may directly affect BASDAI and BASFI scores. The BASMI score consists of four components: spinal flexion, lateral flexion, cervical rotation, and intermalleolar distance, and primarily reflects structural damage to the spine and hip joints. However, the pattern of spinal and hip joint structural damage in SAPHO patients may differ from that in AS patients. Therefore, the correlation between SPARCC scores and BASFI/BASMI in SAPHO patients observed in this study should be interpreted with caution.

## Conclusion

Our study confirms that SAPHO syndrome and AS exhibit a high degree of similarity in the extent, distribution, and characteristics of BME observed on MRI. This similarity offers a preliminary basis for applying treatment strategies from SpA to SAPHO patients with axial involvement. Compared to AS patients, SAPHO syndrome patients show less extensive BME in the spine and sacroiliac joints, whereas vertebral BME in SAPHO patients may have a certain correlation with functional scores. Considering the substantial overlap in MRI inflammatory patterns, SAPHO syndrome with spinal involvement may share underlying mechanisms with SpA. Further studies are warranted to investigate the common pathogenic features between the two conditions.

## Data Availability

The raw data supporting the conclusions of this article will be made available by the authors, without undue reservation.
